# Enhanced YOLOv7 integrated with small target enhancement for rapid detection of objects on water surfaces

**DOI:** 10.3389/fnbot.2023.1315251

**Published:** 2023-12-14

**Authors:** Jie Yu, Hao Zheng, Li Xie, Lei Zhang, Mei Yu, Jin Han

**Affiliations:** ^1^Hubei Key Laboratory of Intelligent Vision Based Monitoring for Hydroelectric Engineering, School of Computer and Information, China Three Gorges University, Yichang, China; ^2^School of Computer and Information, China Three Gorges University, Yichang, China; ^3^State Grid Yichang Electric Power Supply Company, Yichang, China

**Keywords:** object detection, water target detection, YOLOv7, unmanned surface vessel, small object detection

## Abstract

Unmanned surface vessel (USV) target detection algorithms often face challenges such as misdetection and omission of small targets due to significant variations in target scales and susceptibility to interference from complex environments. To address these issues, we propose a small target enhanced YOLOv7 (STE-YOLO) approach. Firstly, we introduce a specialized detection branch designed to identify tiny targets. This enhancement aims to improve the multi-scale target detection capabilities and address difficulties in recognizing targets of different sizes. Secondly, we present the lite visual center (LVC) module, which effectively fuses data from different levels to give more attention to small targets. Additionally, we integrate the lite efficient layer aggregation networks (L-ELAN) into the backbone network to reduce redundant computations and enhance computational efficiency. Lastly, we use Wise-IOU to optimize the loss function definition, thereby improving the model robustness by dynamically optimizing gradient contributions from samples of varying quality. We conducted experiments on the WSODD dataset and the FIOW-Img dataset. The results on the comprehensive WSODD dataset demonstrate that STE-YOLO, when compared to YOLOv7, reduces network parameters by 14% while improving AP50 and APs scores by 2.1% and 1.6%, respectively. Furthermore, when compared to five other leading target detection algorithms, STE-YOLO demonstrates superior accuracy and efficiency.

## 1 Introduction

Unmanned surface vessel (USV) are now widely used in the fields of harbor surveillance, fisheries monitoring, maritime management, and military intelligence analysis, such as target detection and environmental monitoring (Zhang et al., 2022, 2023; Zhou et al., 2022). Equipped with cameras, lasers, and an array of sensors, USVs enable autonomous detection and recognition of their surroundings. This capability ensures the safe and efficient navigation of unmanned vessels, while also enhancing the effectiveness of monitoring tasks. Target detection technology plays a pivotal role in evaluating the performance of unmanned vessels for object detection and recognition tasks. Deep learning-based target detection algorithms have garnered significant attention due to their precision in identifying objects. However, the high-speed mobility of unmanned vessels introduces considerable scale variations in the objects detected. This demands robust multi-scale detection capabilities from the algorithm.

At present, several researchers are actively delving into the realm of waterborne target detection. Moosbauer et al. (2019) publicly released the Singapore Maritime Dataset (SMD), which has provided invaluable resources to drive progress in the field of sea surface target detection from a horizontal perspective. Shin et al. (2020) pioneered the utilization of instance segmentation techniques to extract ship targets from the SMD dataset. These targets were subsequently merged with ocean backgrounds, resulting in a synthetic dataset that significantly enhances the precision of sea surface target detection. Chen et al. (2018) curated a dataset comprising 1,500 images of sea surface targets, drawing from three distinct sources: MS COCO (Lin et al., 2014), Pascal VOC (Everingham, 2010), and SMD. This dataset played a pivotal role in validating their proposed hierarchical, multi-scale deep convolutional neural network-based sea surface target detection algorithm. Furthermore, Zhou et al. (2021) have contributed to the field by curating the WSODD dataset, which stands as a notable advancement in the domain of water target detection, Cheng et al. (2021) collected a variety of local floating garbage to form the FloW dataset—the world's first unmanned ship-view of floating garbage detection dataset, promoting the rapid development of floating garbage detection technology.

**Figure 1:**
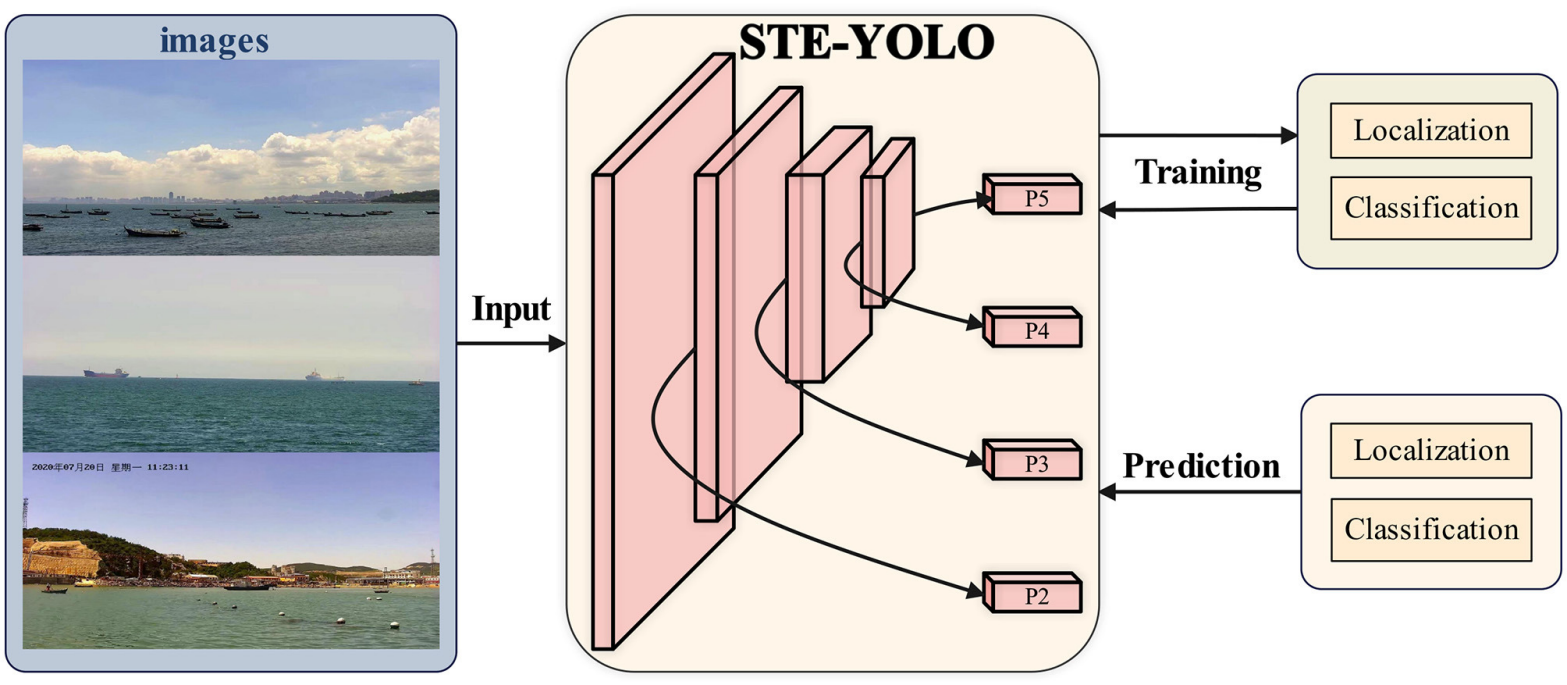
The overview of working pipeline using STE-YOLO. Compared to original version, we mainly improve the method by adding one more head (P2) to better detect different scale objects. In addition, we also employ LVC and L-ELAN to make STE-YOLO stronger.

To address the limitations of existing water target detection algorithms, particularly in the realms of multi-scale and small target detection, we present STE-YOLO, an enhanced model built upon the foundation of YOLOv7. The overview of the detection pipeline utilizing STE-YOLO is depicted in Figure 1. This framework achieves a reduction in network size by meticulous redesign and optimization of the network structure. Additionally, it integrates multi-scale and multi-level information, augmenting the network capacity to characterize objects effectively. Moreover, a residual module is incorporated to heighten the model sensitivity to small targets. The contributions of this paper are delineated as follows:

1. We establish a specialized detection head, focused on precisely detecting small targets. This strategic design empowers the network to adeptly leverage shallow-level information, thereby enhancing its efficacy in identifying small targets. Consequently, this improvement extends to the network's overall capability to detect targets across diverse scales.
2. The introduction of the lite visual center (LVC) module seamlessly merges coordinate convolution with target-relative positional information within the network architecture. This fusion facilitates refined feature extraction from the target region, thereby intensifying attention and precision in detecting small targets. This integration culminates in an elevated overall performance in target detection.
3. The integration of the lite efficient layer aggregation networks (L-ELAN) module into the network results in reduced parameters and operations, all while upholding accuracy.
4. Optimization of the loss function computation is achieved through the utilization of Wise-IOU (Tong et al., 2023), thereby boosting the model confidence level and enhancing its robustness.

## 2 Related works

### 2.1 Object detection based on deep learning

Modern deep learning-based methods for target detection can be categorized into two primary types: two-stage target detectors and single-stage target detectors. Two-stage target detectors, represented by architectures like Mask-RCNN (He et al., 2017), VFNet (Zhang et al., 2021), and CenterNet (Zhou et al., 2019), generate a set of region proposals using a region selection network. These proposals then undergo feature extraction through a learning module, followed by classification and regression processes. However, this method of extracting features for each proposal can lead to significant computational costs and might not capture a comprehensive global feature representation effectively. On the other hand, prevalent single-stage detectors, exemplified by networks like YOLOX (Ge et al., 2021), FCOS (Tian et al., 2022), Scaled-YOLOv4 (Wang et al., 2021), and EfficientDet (Tan et al., 2020), rely on a backbone network to extract feature maps for the entire input image. These feature maps are subsequently used to predict bounding boxes, enabling concurrent prediction and classification through box generation.

Furthermore, in terms of the network's architectural components, it can be divided into two primary elements: the Backbone, responsible for extracting image features, and the Head, utilized for predicting object categories and bounding box coordinates. Additionally, some researchers have introduced an intermediary module known as the Neck between the Backbone and the Detection Head to optimize detection performance.

**Backbone**. Commonly utilized backbone networks include ResNet (Wightman et al., 2021), CSPDarknet53 (Bochkovskiy et al., 2020), Swin Transformer (Liu et al., 2021), and FasterNet (Chen J. et al., 2023). These backbones exhibit robust feature extraction capabilities, particularly for classification tasks. Typically, researchers only need to fine-tune the backbone to optimize its performance for specific tasks.

**Neck**. The Neck network is designed to enhance the utilization of features derived from the Backbone network. It accomplishes this by reprocessing the feature maps extracted by the Backbone at various stages. The Neck network typically consists of multiple bottom-up and top-down pathways. What sets this network apart is its direct multi-stage feature mapping approach, which omits feature layer aggregation operations and aligns directly with the Head. Prominent Neck network architectures include PANet (Wang et al., 2019), NAS-FPN (Ghiasi et al., 2019), and SFAM (Zhao et al., 2019). These architectures often utilize combinations of up-sampling and down-sampling iterations, concatenation, element-wise summation, and dot products to establish effective aggregation strategies. Additionally, complementary modules like MSFFM (Wan et al., 2023), ASPP (Weber et al., 2021), SPPCSPC (Wang et al., 2023), and GFFAP (Sun et al., 2021) are incorporated into Neck networks to enhance feature fusion and improve detection accuracy. Adding an attention module is also a great way to do this, such as MFINEA (Sun et al., 2023) and Box-Attention (Nguyen et al., 2022).

**Head**. While the backbone network primarily functions as a classification network, it is insufficient to perform the localization task independently. Hence, the head network plays a crucial role in achieving both target localization and categorization, utilizing the feature maps extracted by the backbone network. Head networks are generally divided into two main categories: single-stage detectors and two-stage detectors (He et al., 2017; Ren et al., 2017). stands out as a notable representative of two-stage detectors. On the other hand, one-stage detectors offer faster prediction by simultaneously estimating bounding boxes and target categories, but they may sacrifice accuracy. Due to the real-time constraints in water target detection, a majority of algorithms used in this domain opt for one-stage detectors, such as YOLO (Wang et al., 2023) and SSD (Zalesskaya et al., 2022).

### 2.2 Small target detection

Small target detection presents significant challenges within the realm of target detection, necessitating algorithms with robust fine feature extraction capabilities (Shamsolmoali et al., 2022; Gong, 2023). Typically, two primary criteria are employed to define small targets: absolute size and relative size. In terms of absolute size, targets with dimensions smaller than 32 × 32 pixels are categorized as small. In the case of relative size, targets with an aspect ratio <0.1 times the original image size are considered small (Lin et al., 2014). Currently, small target detection algorithms fall into three main categories: those utilizing data augmentation, those emphasizing multi-scale learning, and those leveraging contextual information.

**Data augmentation**. Kisantal et al. (2019) elevated the percentage of small targets within the dataset through replication, thereby enhancing their significance in the network. This augmentation aimed to bolster the model's proficiency in detecting small targets. Yu et al. (2020) introduced the scale-matching strategy, aligning pre-trained network features with those obtained by the detector. This strategy ensures the comprehensive utilization of pre-trained network capabilities, thereby enhancing overall performance.

**Multiscale learning**. The deep network has large receptive field and strong representation ability of semantic information, but weak representation ability of geometric information. The lower layer network has relatively small receptive field and strong representation ability of geometric details, but weak representation ability of semantic information. Thus, Multiscale learning often enhances network representation ability by fusing shallow detail information with deep semantic information, thus improving small target detection. However, multiscale learning can increase parameters and slow down inference speed. The Feature Pyramid Network (FPN), proposed by Lin et al. (2017), is a classic multiscale learning network structure. In FPN, the image undergoes bottom-up feature extraction, followed by top-down feature fusion, before being fed into the detection head for regression prediction. Deng et al. (2022) extended this approach with the Enhanced Feature Pyramid Network (EFPN), which incorporates a feature texture migration module for ultra-high-resolution feature extraction, further enhancing small target detection.

**Utilization of contextual information**. Zhu et al. (2021) introduced TPH-YOLOv5, a novel strategy that integrates the Transformer (Vaswani et al., 2017) into the prediction head of YOLOv5 (Jocher et al., 2022). This integration enhances predictive regression capabilities while employing an attention mechanism to focus intensively on small targets. In a different vein, QueryDet (Yang et al., 2022) utilizes a querying mechanism to expedite target detector inference. It leverages low-resolution features for preliminary localization predictions, which then guide higher-resolution features, contributing to increased accuracy in predictive regression.

## 3 STE-YOLO

YOLOv7 (Wang et al., 2023) stands out as a highly proficient one-stage detector, renowned for its exceptional overall performance. The model structure comprises three central components: Backbone, Neck, and Head. The YOLOv7 framework incorporates a wide range of advanced techniques, substantially enhancing its detection capabilities. Operating as a one-stage detector, YOLOv7 boasts impressive real-time processing capabilities, rendering it particularly well-suited for meeting the demands of real-time water-based target detection.

**Figure 2:**
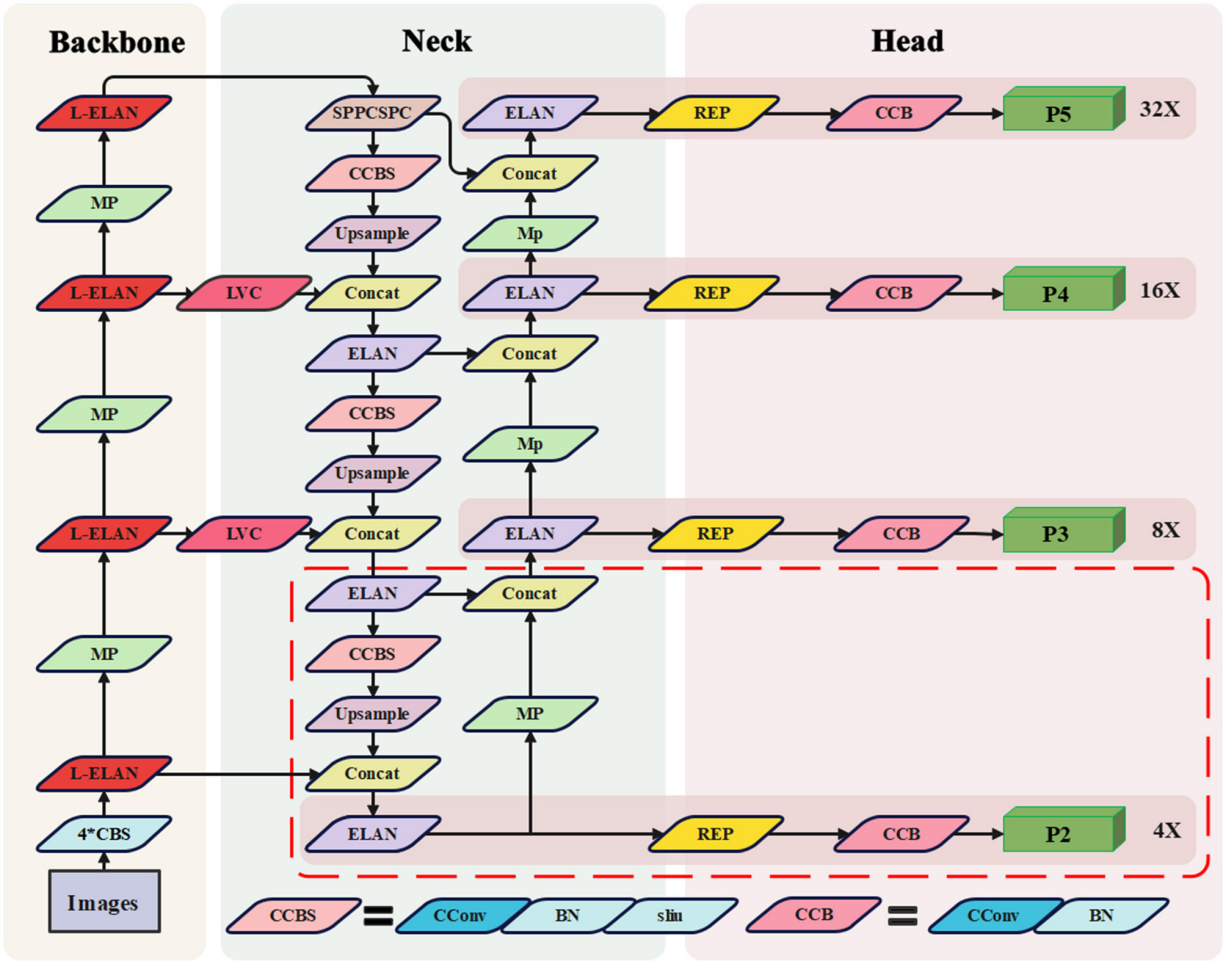
Architecture of STE-YOLO. CSPDarknet53 backbone with four L-ELAN blocks. The Neck use the structure like PANet with LVC. Four prediction heads use the different size of feature maps Neck. The CBS module is a basic module, which contains three operations of convolution, normalization and activation function, the CConv means CoordConv.

However, due to the susceptibility of water target detection to adverse weather conditions like storms, coupled with the high-speed nature of USVs leading to significant variations in target scale, YOLOv7 encounters certain challenges. These include limitations in recognizing targets across diverse scales, which in turn reduces accuracy in rapidly detecting small targets in water scenarios. With a focused approach, our aim is to enhance YOLOv7's proficiency in detecting small targets while optimizing computational efficiency. Our proposed solution is a waterborne rapid target recognition algorithm named STE-YOLO. The architectural diagram, illustrated in Figure 2, introduces a novel detection branch known as P2 head, represented by dashed lines. The P2 head primarily enhances the detection performance of small targets while simultaneously accommodating multi-scale target detection. The LVC module employs coordinate convolution to amplify focus on small targets, replacing the original fusion operation in the FPN structure and amalgamating multi-level information to enhance detection accuracy. Additionally, the L-ELAN module, a lightweight feature extraction addition, aims to reduce network computation while bolstering network robustness and computational efficiency. Furthermore, we have fine-tuned the loss function and integrated Wise-IOU to achieve improved detection performance.

### 3.1 Small target head P2

**Figure 3:**
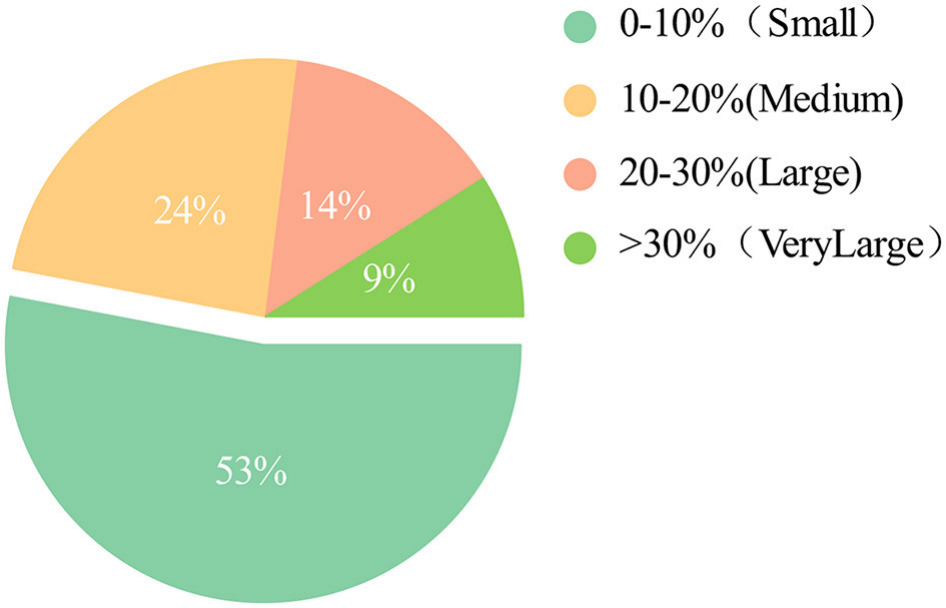
Percentage of targets of different sizes in the WSODD dataset.

In the context of actual waterborne target detection tasks, encountering a substantial volume of small target samples (Zhou et al., 2021) is common. Through in-depth data analysis of the prevalent water target dataset WSOOD, a notable insight has emerged. This dataset comprises an impressive 53% of small targets, as depicted in Figure 3. Consequently, the task of waterborne target detection demands that the model possesses a strong capability to extract localized information, persisting across the intricate layers of the network. This is crucial to ensure the comprehensive retention of information relevant to small targets. YOLOv7 adheres to the downsampling mechanism typical of conventional convolutional networks, where increasing depths lead to augmented downsampling factors. This mechanism fosters a wider spatial perception, which is advantageous for recognizing overarching target contours. However, this approach also carries the risk of losing detailed information, which could significantly hinder small target recognition.

**Figure 4:**
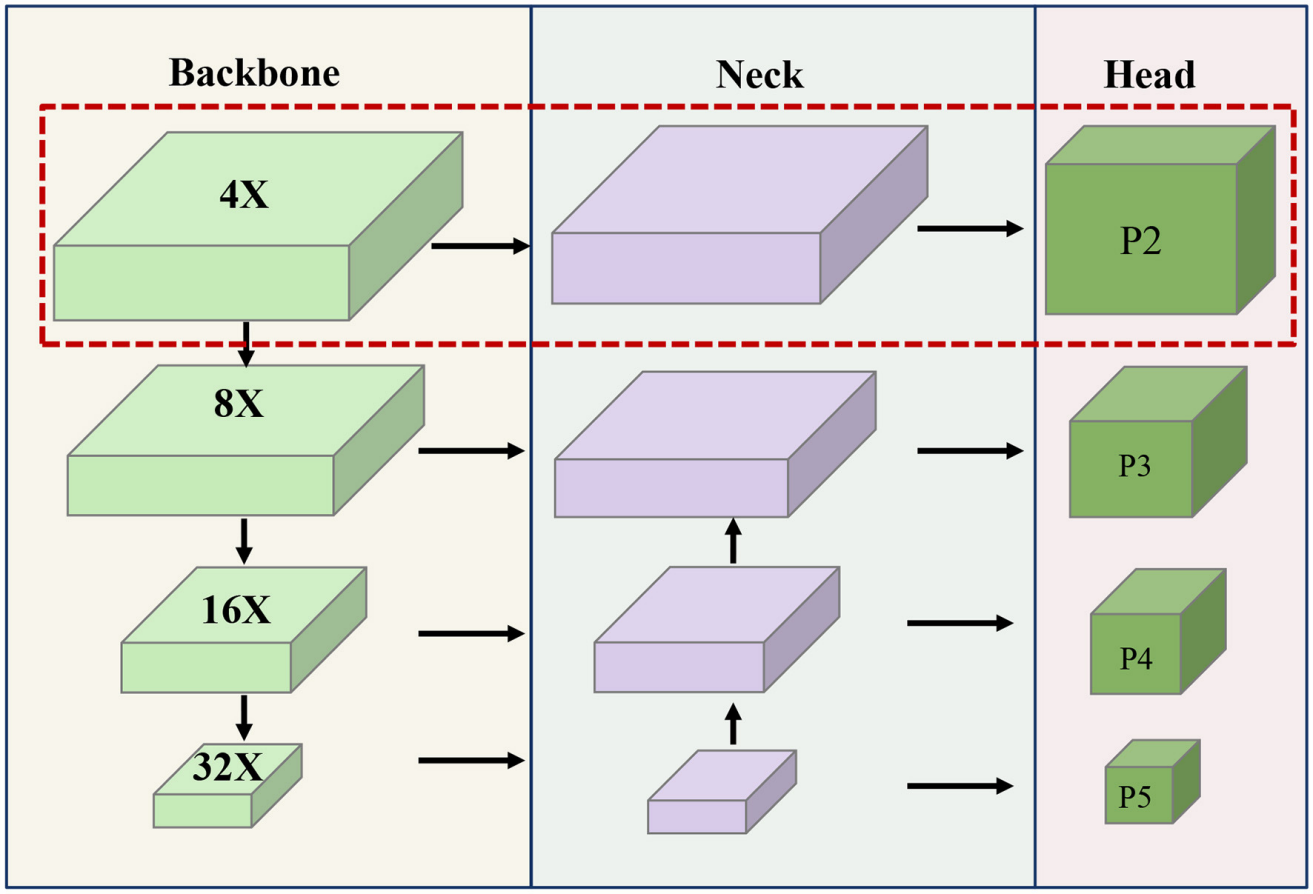
The overview of Head network optimization results, the portion of the red box is the newly added P2 detector head.

With this challenge in mind, we have developed a specialized prediction head network called P2 head that places a primary focus on recognizing small targets. As an innovative extension, a shallow feature obtained through a 4× downsampling rate is introduced as one of the inputs to the neck network. Subsequently, the neck network orchestrates the fusion of four distinct sets of features, each corresponding to varying scales downsampling rates of 4×, 8×, 16×, and 32×. These amalgamated features are then directed to the head network, resulting in the creation of four distinct detector head structures. Each of these structures is optimized for detecting objects of different sizes. The architectural arrangement is vividly depicted in Figure 4.

The newly introduced P2 detection branch is meticulously crafted for the explicit purpose of detecting exceedingly small targets. Given the inherent possibility of significant information loss in deeper feature maps due to consecutive convolutional pooling, and the risk of larger target features overpowering those of smaller targets, a challenge of misdetection and omission emerges. As a response, there is a proactive approach in place for the input to the P2 detection branch structure, predominantly originating from the shallow convolutional layer. This particular layer encapsulates a wealth of localized information, spanning attributes such as shape, position, and size. Consequently, it greatly assists in precisely localizing small targets. This strategic enhancement effectively bolsters the efficiency of small target detection, all the while catering to the broader capabilities of multi-scale target detection. Furthermore, this architectural extension comprises four distinct detector head structures, each serving as a mitigation strategy against the adverse consequences of significant variations in target scale. This configuration, in turn, ultimately contributes to the elevation of the comprehensive detection performance.

Furthermore, YOLOv7 exhibits a high degree of adaptability to the configuration of the anchor frame dimensions. Therefore, when utilizing the P2 detection branch for predictive regression, meticulous assessment of the anchor frame dimensions becomes imperative. This evaluation involves a tailored K-means cluster analysis that aligns with the dataset's characteristics. Its goal is to determine the most suitable anchor frame size. Once this determination is made, the anchor frame settings for each branch are established, as outlined in Table 1. As depicted in Table 1, the P2 detection branch holds the potential to address scenarios where the target object might evade detection due to its diminutive size combined with an excessively large anchor frame. The strategic alignment effectively alleviates challenges of misdetection and omission that may arise from suboptimal anchor frame configurations P3 head. This approach ensures a robust and reliable detection process by fine-tuning the anchor frame settings to harmonize with the unique characteristics of the detection task.

**Table 1:** The head setting of STE-YOLO.

**Prediction head**	**Anchor box setting**
P2	[8,10, 9,22, 17,16]
P3	[10,13, 16,30, 33,23]
P4	[30,61, 62,45, 59,119]
P5	[116,90, 156,198, 373,326]

### 3.2 Lite visual center–LVC

Given the intricate and ever-changing nature of the aquatic environment, a multitude of dynamic factors come into play. These factors encompass light reflections, precipitation, interference induced by fog, and the constant fluctuations in wind and wave patterns. These phenomena often converge to create challenging conditions. These conditions, frequently presenting as shadows, reflections, or image blurring, severely compromise the visibility of targets. Consequently, the intricacies associated with target identification become more pronounced. Therefore, there is a compelling imperative to devise innovative and efficient strategies for feature extraction. These strategies are specifically designed to enhance the capacity for acquiring both broad context and localized information. This strategic endeavor assumes paramount importance in the pursuit of heightening the overall effectiveness of detection.

**Figure 5:**
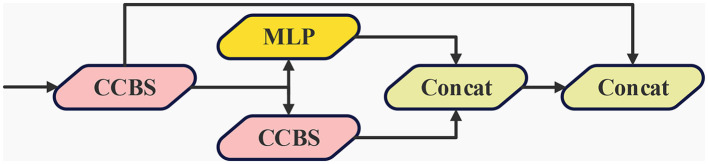
The architecture of LVC, which contains two main blocks, a Multilayer Perceptron (MLP) and CCBS, residual paths are also used.

While YOLOv7 employs a singular convolutional fusion to link the backbone network with the neck network, effectively facilitating the extraction and fusion of features across diverse levels and thereby enhancing the capability for multi-level feature extraction to some extent, its adequacy in recognizing small targets falls short. This insufficiency gives rise to the introduction of the lite visual center (LVC) module. This module is inspired by the structural blueprint of EVC (Quan et al., 2023), which is present within the framework of CFP. The architectural details of this novel module are visually represented in Figure 5.

Within the LVC module, two critical components are seamlessly integrated the multilayer perceptron [MLP (Tolstikhin et al., 2021)] and CCBS [CoordConv (Liu et al., 2018) with bilateral strategy]. The incorporation of the MLP component serves a pivotal role in capturing extensive global long-term dependencies within deep features, which results in the encapsulation of global information and ultimately enhancing the precision of holistic recognition. Concurrently, the introduction of CCBS involves the application of CoordConv, a convolutional mechanism enriched with relative position coordinate information during the convolution process. This integration empowers the network to discern and ascertain the relative positioning of targets more effectively through the amalgamation of localized area features. Ultimately, the feature maps originating from the MLP and CCBS modules are amalgamated across the channel dimension, thus constituting the output of the LVC module. This amalgamated feature suite seamlessly integrates the strengths of both modules, preserving significant information concerning elements within the block or pool, and capable of supplying more comprehensive and enriched multi-level fusion features. Consequently, the detection model is able to acquire a holistic range of feature representations, thereby amplifying the recognition capacity for small targets.

Simultaneously, this paper undertakes the replacement of selected CBS modules in the original model with CCBS modules, allowing the network to harness the amalgamated positional coordinate data and global information, ultimately enhancing the overall system performance. These innovative addition serves as a means to further enrich the model feature extraction capabilities, especially in scenarios where small targets play a pivotal role.

### 3.3 L-ELAN

**Figure 6:**
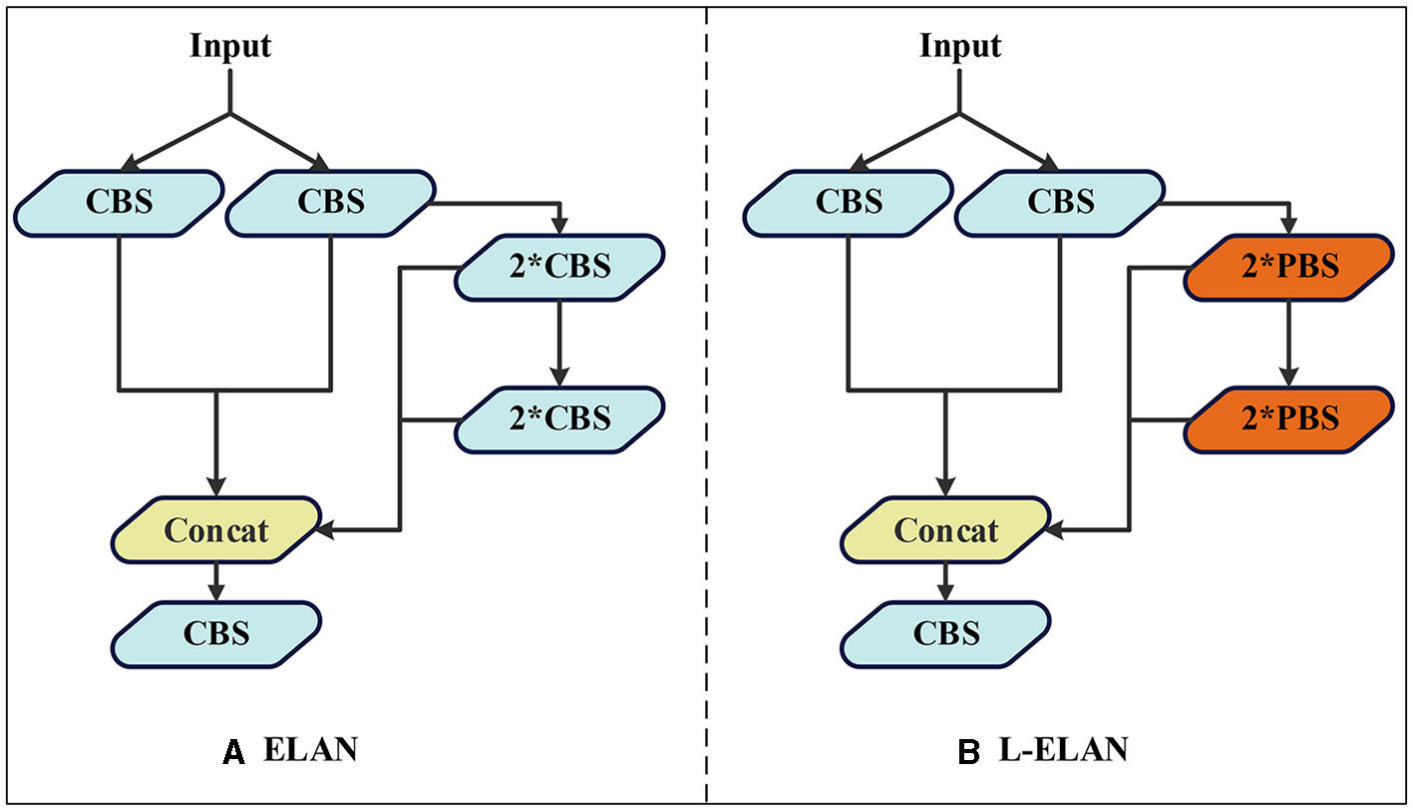
The structure of ELAN **(A)** and L-ELAN **(B)**, where PBS modules are combinations of Pconv, BN, and silu activation functions, and all PBS modules in the ELAN structure are CBS.

In the realm of water target detection tasks, the inherent limitations of equipment often require the downsizing of the model for enhanced practical applicability. With this consideration, a L-ELAN lightweight module is put forth, meticulously striking a balance between model accuracy and computational efficiency. The schematic representation of this module can be observed in Figure 6. The water target detection task, due to the limitation of equipment, it is often necessary to reduce the model size as much as possible for ease of use. For this reason, a lightweight module L-ELAN is proposed by considering model accuracy and computational efficiency, as shown in Figure 6.

**Figure 7:**
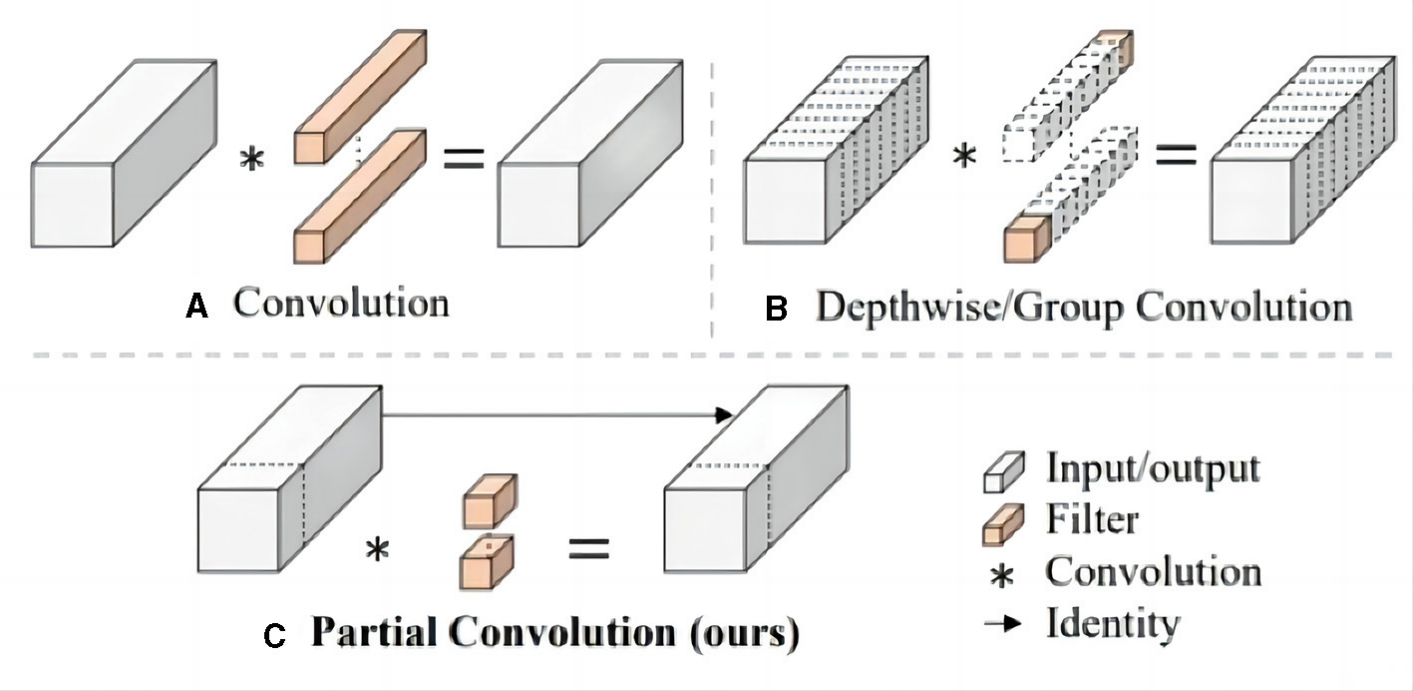
The structure of Conventional convolution **(A)**, Dconv **(B)**, and Pconv **(C)**, the Pconv is only applied to a part of the input channel for spatial feature extraction without a affecting the rest of the channels.

In the original YOLOv7 framework, the efficient layer aggregation networks (ELAN) module predominantly employs conventional convolutions for feature extraction. While this methodology ensures a commendable level of detection accuracy, it doesn't inherently excel in terms of computational efficiency. In response to this challenge, the lite efficient layer aggregation networks (L-ELAN) module is introduced. It integrates positional convolution [PConv (Chen J. et al., 2023)] to replace specific segments of the traditional convolution processes The structure of PCONV is shown in Figure 7. This strategic substitution leverages the inherent traits of PConv minimal computational requirements and heightened efficiency. The integration of PConv within L-ELAN serves a dual purpose: enhancing the network computational efficiency and reducing the overall count of network parameters. This innovative enhancement aligns with the overarching goal of optimizing the network performance not only in terms of detection accuracy but also in terms of computational resource utilization. By strategically selecting and incorporating PConv within the L-ELAN module, the model achieves a balance between accuracy and efficiency a crucial consideration in real-time waterborne target detection scenarios.

When handling input **I**∈ℝ^*c*×*h*×*w*^, conventional convolution utilizes *c* filters **W**∈ℝ^*k*×*k*^ to perform computations and generate an output **O**∈ℝ^*c*×*h*×*w*^. In contrast, PConv employs standard convolution for spatial feature extraction exclusively on select input channels, while leaving the remaining channels unaffected. This methodology ensures an alignment between the number of channels in the input and the resulting output feature maps. The computational cost, quantified in terms of FLOPS (Floating Point Operations Per Second), associated with the standard convolution operation is as follows:


(1) $$\begin{array}{l} FLOPs=h\times w\times k^{2}\times c \end{array}$$

where *h*, *w* is the size of the feature map, *k* is the size of the filters, *c* is the number of filters used. The FLOPS calculation formula for PConv is as follows:


(2) $$\begin{array}{l} FLOPs=h\times w\times k^{2}\times c_{p}^{2}. \end{array}$$

where *c*_*p*_ is the number of partial channels. When the convolution channel percentage $r=\frac{c_{p}}{c}=\frac{1}{4}$, the FLOPS of PConv is just $\frac{1}{16}$ of the traditionally convolution.

Furthermore, PConv exhibits reduced memory accesses (*M*), which can be computed as demonstrated in Eq. 3, while the conventional convolution is represented by Eq. 4.


(3) $$\begin{array}{l} M=h\times w\times 2c_{p}+k^{2}\times c_{p}^{2}\approx h\times w\times 2c_{p} \end{array}$$

(4) $$\begin{array}{l} M=h\times w\times 2^{c}+k^{2}\times c^{2}\approx h\times w\times 2c \end{array}$$

The computational effort is merely a quarter of that required by the normal convolution when *r* equals $\frac{1}{4}$.

The analysis provided above leads to a clear realization that the incorporated L-ELAN module within this study achieves two pivotal objectives. Primarily, it significantly reduces the computational load while simultaneously effectively addressing memory access demands. This harmonious accomplishment notably streamlines and expedites the practical feasibility of real-world deployment.

### 3.4 Wise-IOU

In the realm of water target recognition tasks, it's commonplace to encounter a notable prevalence of suboptimal samples, particularly within datasets that display limitations and imbalances (Chen X. et al., 2023). After thorough examination, this study establishes that the CIOU loss function, when integrated into the original YOLOv7 model, exerts a substantial negative impact on the cumulative regression loss. This issue predominantly arises from samples characterized by subpar regression quality. Concurrently, it introduces difficulties in effectively fine-tuning samples that possess relatively higher regression quality, thereby compromising the overall effectiveness of recognition. To navigate these challenges, this paper deliberately chooses to adopt the Wise-IOU loss function (WIOUv3 version), as outlined in Eq. 5.


(5) $$\begin{array}{l} L_{WIOU_{v3}}=rR_{WIOU}L_{IOU} \end{array}$$

where $L_{IOU}\in \left(0,1\right)$ is the ratio of the intersection of the prediction frame and the true frame, *R*_*WIOU*_∈[1, *e*) means distance attention, *r* is non-monotonic focusing factor, which can adaptively adjust the gradient gain assignment strategy according to the degree of outliers of the anchor frame.

The Wise-IOU loss function incorporates a dynamic non-monotonic focusing mechanism, leveraging “outliers” in place of IOUs for the assessment of anchor frame quality. This approach also encompasses an intelligent strategy for assigning gradient gains. The expressions for *R*_*WIOU*_ and *r* are outlined below:


(6) $$\begin{array}{l} R_{WIOU}=\exp\left(\frac{{\left(x-x_{gt}\right)}^{2}+{\left(y-y_{gt}\right)}^{2}}{{\left({W_{g}}^{2}+{H_{g}}^{2}\right)}^{*}}\right) \end{array}$$

(7) $$\begin{array}{l} r=\frac{\beta }{\delta \alpha^{\beta -\delta }} \end{array}$$

where *x* and *y* denote the anchor frame dimensions in terms of length and width, while *W*_*g*_ and *H*_*g*_ represent the dimensions of the minimum enclosing frame. The symbol (*) serves to mark the detachment of *W*_*g*_ and *H*_*g*_ from the computational graph, effectively excluding them from participating in backpropagation.

This measure is undertaken to avoid the emergence of gradients that might hinder convergence, particularly concerning the *R*_*WIOU*_ computation. β assumes significance as the outlier value, functioning as a descriptor for the anchor frame quality. δ and α take on the role of hyperparameters This description takes the following form.


(8) $$\begin{array}{l} \beta =\frac{L_{IOU}^{*}}{L_{IOU}}\in \left[0,+\infty \right) \end{array}$$

Due to the small data set, we hope that the model can reach the high gradient gain earlier in the training process, so we choose to increase and decrease to improve the speed of reaching the peak, so that the model anchor frame can obtain the highest gradient gain earlier. The final experimental results also prove this.

In our established framework, a smaller departure from the norm signifies an anchor frame boasting heightened quality. Consequently, these high-quality anchor frames receive minimal gradient gains, directing the emphasis of bounding box regression toward anchor frames of average quality. Conversely, anchor frames exhibiting larger deviations are coupled with relatively subdued gradient gains, effectively countering the emergence of unfavorable gradients originating from subpar samples. This strategy not only tempers the influence wielded by high-quality anchor frames but also mitigates the adverse gradients arising from low-quality instances. This carefully calculated approach serves to harmonize the weighting assigned to a diverse spectrum of samples, steering attention toward anchor frames that epitomize an intermediate standard. This intentional focal point on anchor frames with moderate performance significantly fortifies the model resilience, ultimately yielding a conspicuous enhancement in the overall performance of the detection system.

## 4 Results

### 4.1 Implementation details

In the scope of this research, experimentation unfolds within the Ubuntu environment, specifically the 18.04.6 version. Both training and testing occur on a sole NVIDIA RTX3090 GPU. The chosen deep learning framework is PyTorch 1.10.1, harnessed alongside CUDA version 11.7. The model foundational weights stem from prior training on the COCO dataset. The encompassing training regimen spans 200 epochs, commencing with an initial learning rate of 0.01, which subsequently tapers to 0.001 in the concluding cycle set banchsize to 1 when calculating FPS. The training pipeline leverages the Stochastic Gradient Descent (SGD) optimization technique, with the incorporation of momentum, anchored at 0.937. Simultaneously, the weight decay coefficient stands at 0.0005. Importantly, the model operates on a consistent scale of input images, each adopting dimensions of 640× 640 pixels, while each training batch encompasses 12 samples.

Within this study, we use the WSODD dataset Zhou et al. (2021), which is commonly used in water target detection, and consists of images from oceans, lakes, and rivers with different climatic conditions and shooting times, with a total of 7,467 images, and the resolution of each image is 1,920*1,080. In addition, the dataset consists of a total of 14 common object classes and 21,911 instances . In this paper, it is divided into training set, testing set, and validation set, and the performance of the model is evaluated with reference to the criteria of COCO dataset (Lin et al., 2014).

We also used the FIOW-Img subdataset Cheng et al. (2021), which is the world's first floating garbage detection dataset in a real inland river scene from the perspective of an unmanned ship. The FloW-Img subdataset contains 2,000 images and 5,271 marked targets. One thousand two hundred images are randomly selected as the training set and the rest as the verification set and test set. Small targets (size in 32× 32) in this dataset account for a large proportion (about 60%), which is beneficial for testing the performance of relevant algorithms on small targets.

### 4.2 Ablation studies

To assess the efficacy of the proposed P2 head, LVC, L-ELAN, Wise-IOU presented in this paper, we conducted a series of comparative experiments. The outcomes of these experiments are detailed in Table 2.

**Table 2:** Ablation study of proposed method on WSODD test dataset.

**Methods**	**AP50 (%)↑**	**FLOPs (G)↓**	**Para (M)↓**
YOLOv7	81	103.4	34.56
YOLOv7 + P2	81.9 (↑0.9)	107.5 (↑4.2)	36.07 (↑1.21)
YOLOv7 + LVC	81.7 (↑0.7)	105.6 (↑2.3)	35.89 (↑1.03)
YOLOv7 + L-ELAN	81.3 (↑0.3)	83.1 (↓20.3)	30.56 (↓4.3)
YOLOv7 + Wise-IOU	81.6 (↑0.6)	103.4 (−)	34.86 (−)
STE-YOLO	83.1 (↑2.1)	89.6 (↓13.8)	32.8 (↓2.06)

↑ Means the larger the better, ↓ means the smaller the better.

#### 4.2.1 P2 head

The integration of the P2 Head to enhance small object detection slightly increases the network parameters. However, this adjustment corresponds to a notable 0.8% enhancement in the AP value. This outcome underscores the beneficial influence of the P2 Head in bolstering the detection of small targets.

#### 4.2.2 LVC

Upon incorporation of the LVC module, the computational complexity of the model in this study registers a 2% growth in GFLOPs and a 1% increase in parameters. Remarkably, the AP50, a key performance metric, experiences a substantial 0.9% improvement. This observation substantiates the effectiveness of the LVC module in significantly enhancing target recognition rates.

#### 4.2.3 L-ELAN

Upon activation of the L-ELAN module, the GFLOPs of the model in this paper reduce from 103.4 to 83.1, accompanied by a parameter reduction from 34.86 to 30.56. This translates to a reduction of 20% and 13% respectively. Notably, this reduction in computational demands is accompanied by a 0.3% increase in AP. The implications of these findings are twofold: the L-ELAN module not only achieves an effective reduction in network size, but also plays a pivotal role in elevating the accuracy of target detection.

#### 4.2.4 Further validation of L-ELAN

To further substantiate the performance of the L-ELAN module, a comparative evaluation involving distinct convolutional modules (Yang et al., 2019), (Zhu et al., 2019) is carried out. The ensuing results are detailed in Table 3. Notably, the use of the PConv module leads to an improvement in target detection performance, coupled with lower GFLOPs and a reduced parameter count.

**Table 3:** Comparison of the performance in L-ELAN module.

**Methods**	**AP50 (%)↑**	**FLOPs (G)↓**	**Para (M)↓**
YOLOv7	81	103.4	34.56
Conv	81	103.4	34.56
DCNv2	80.8 (↓ 0.2)	89.1 (↓ 14.3)	36.07 (↑ 1.40)
DCNv3	81.1 (↑ 0.1)	81.1 (↓ 17.9)	35.89 (↓ 1.97)
PConv	81.3 (↑ 0.3)	83.1 (↓ 20.3)	30.56 (↓ 4.30)

↑ Means the larger the better, ↓ means the smaller the better.

#### 4.2.5 Wise-IOU

As evidenced by the data in Table 2, the incorporation of the novel loss function Wise-IOU results in unchanged model parameters and computational operations. However, owing to the influence of the dynamic non-monotonic focusing mechanism, the model described in this paper exhibits a 0.6% enhancement in AP50. This improvement underscores the efficacy of the introduced loss function and its capacity to effectively refine model performance.

### 4.3 Comparisons with the state-of-the-art

To comprehensively validate the overall detection prowess of the model presented in this paper, a thorough comparison is conducted against the benchmark model YOLOv7, along with various cutting-edge target detectors (Carion et al., 2020; Zhu et al., 2020; Jocher et al., 2022; Xu et al., 2022). The ensuing comparative experiments yield results showcased in Tables 4, 5. Notably, the findings in Table indicate that the model proposed in this paper attains the highest score on AP50, along with leading APs and APl scores. This resounding achievement stands as compelling evidence affirming the efficacy of the strategies put forth in this paper.

**Table 4:** The comparison of the performance in WSODD.

**Methods**	**AP50 (%)↑**	**AP95 (%)↑**	**APs (%)↑**	**APm (%)↑**	**APl (%)↑**	**Para (M)↓**	**FLOPS (G)↓**
DETR	79.9	4.60	14.7	34.4	61.8	39.37	86
D-DETR	80.1	38.9	21.1	35.7	56.1	40.01	173
YOLOv5s	79.6	46.3	17.6	37.2	58.3	6.72	15.9
YOLOv5m	80.9	47.3	17.4	38.2	62.0	19.94	48
DAMO-YOLOt	81.3	48.2	20.8	40.3	60.3	8.5	18.2
DAMO-YOLOs	81.6	48.7	19.0	**40.8**	57.5	16.3	37.8
DAMO-YOLOm	81.8	**50.1**	18.7	40.3	61.8	28.2	61.8
YOLOv7t	77.7	46.0	15.5	37.1	60.3	5.78	13.1
YOLOv7m	81.0	49.3	20.3	39.7	62.7	34.86	103.4
YOLOv7x	83.0	49.2	21.6	39.3	62.6	67.62	188.3
STE-YOLO	**83.1**	49.4	**21.9**	39.8	**63.2**	32.8	89.6
YOLOv8n	80.3	47.7	15.8	37.8	62.7	**3.01**	**8.1**
YOLOv8s	81.8	49.2	18.7	38.7	62.5	11.13	28.5
YOLOv8m	82.2	49.9	18.3	39.9	63.0	25.85	78.7

Bold values means the best performance under the same evaluation criteria.

↑ Means the larger the better, ↓ means the smaller the better.

**Table 5:** The comparison of the performance in FIOW-Img.

**Methods**	**AP50 (%)↑**	**AP95 (%)↑**	**APs (%)↑**	**APm (%)↑**	**FPS↑**	**Para (M)↓**	**FLOPS (G)↓**
YOLOv5s	81.1	**38.3**	23.9	52.6	141.5	6.71	15.8
YOLOv5m	81.7	31.1	24.7	53.2	87.1	19.88	47.9
YOLOv5l	82.8	36.4	25.9	55.2	51.3	46.12	107.6
YOLOv7t	80.9	35.7	23.8	53.0	**143.7**	5.76	13.0
YOLOv7	81.7	36.8	24.2	54.1	47.4	34.81	103.2
YOLOv7x	83.0	38.2	25.6	52.4	30.1	67.53	188.0
STE-YOLO	**83.2**	37.3	**26.1**	55.9	56.8	32.74	89.2
YOLOv8n	80.7	30.0	23.5	52.2	125.3	**3.0**	**8.1**
YOLOv8s	81.3	33.4	24.2	55.8	118.7	11.1	28.4
YOLOv8m	81.8	33.5	25.1	**56.3**	92.6	25.77	78.7

Bold values means the best performance under the same evaluation criteria.

↑ Means the larger the better, ↓ means the smaller the better.

When juxtaposed with the benchmark model YOLOv7 on WSODD, our STE-YOLO demonstrates a notable 2.1% enhancement in AP50, as well as a 1.6% improvement in APs, reaching 21.9%. Additionally, the parameter count in this paper model is 14% lower than that of YOLOv7, with a 6% reduction in GFLOPs. Noteworthy is the comparison with DAMO-YOLO and DETR, models with similar GFLOPs. STE-YOLO outperforms both in terms of detection performance. Furthermore, in comparison to the larger YOLOV7x model, the model detailed in this paper reduces parameter count by 53% and GFLOPs by 51%, while simultaneously surpassing AP50 and Aps by 0.1% and 0.3%, respectively.

When the model was tested on the FIOW-Img dataset, our STE-yolo achieved the best results on AP50 and APs, with 1.5% and 1.9% improvements over the original model, respectively, and APs in particular was significantly ahead of the larger version model. In addition, the FPS of this model has also improved, having the highest FPS among models of similar size, reaching 56.8. This also proves the fast detection and optimization effect of the proposed strategy for small targets.

In summary, the STE-YOLO model proposed in this paper demonstrates substantial enhancements across diverse metrics when compared to the standard YOLOv7 model, and it also showcases clear advantages over alternative algorithms in some extent.

### 4.4 Visualization of results

**Figure 8:**
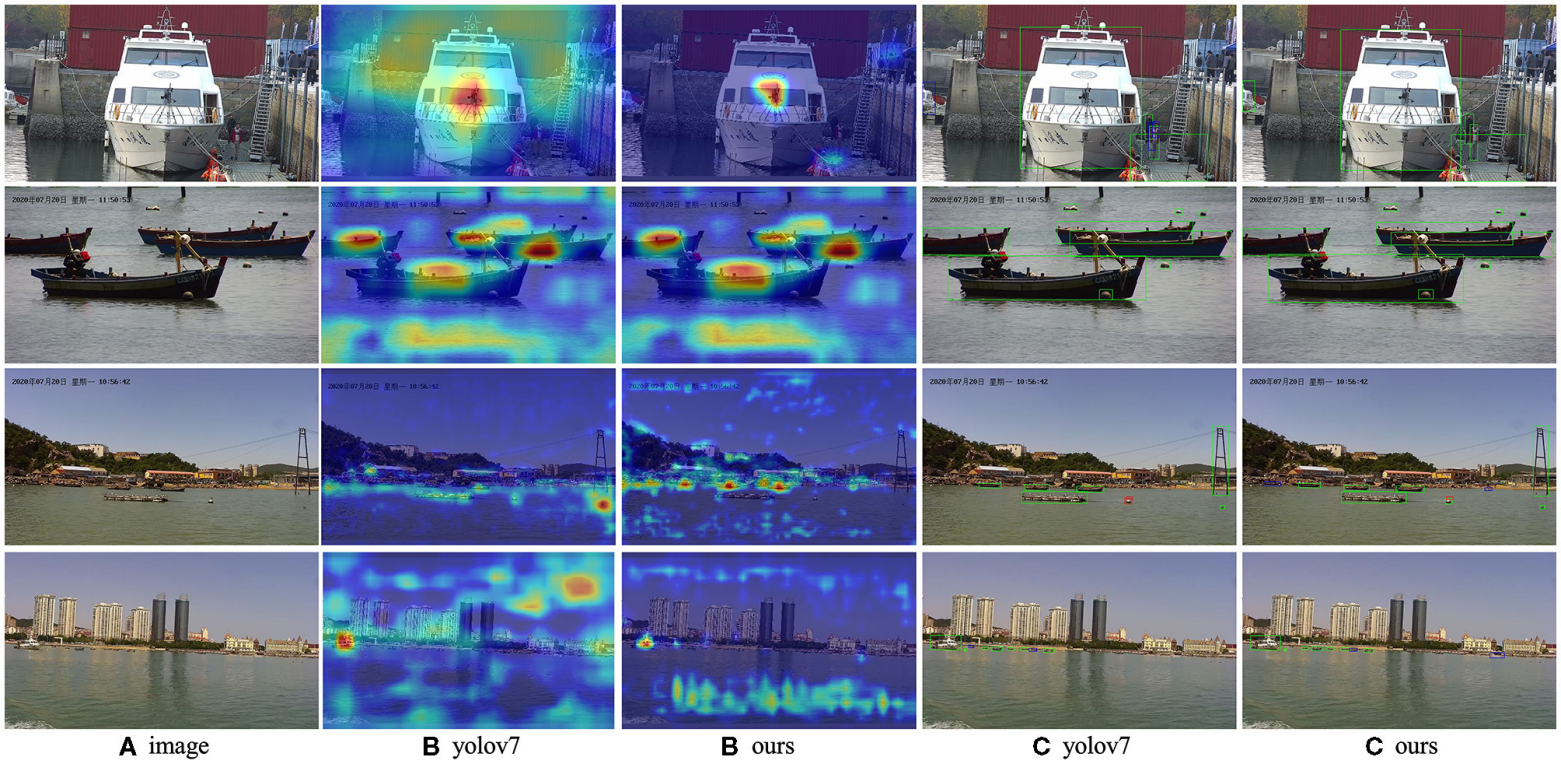
Some visualization results from our STE-YOLO on WSODD. Where **(A)** represents the original picture, **(B)** represents the thermal map, and **(C)** represents the detection map. We are using heat maps and calibration boxes to show the the effect. Use different colors for different test results (green means correct detection, blue means wrong detection, red means missed detection). In our model, the heat map is more centralized, and there are fewer misdetections and omissions compared to the original modele.

**Figure 9:**
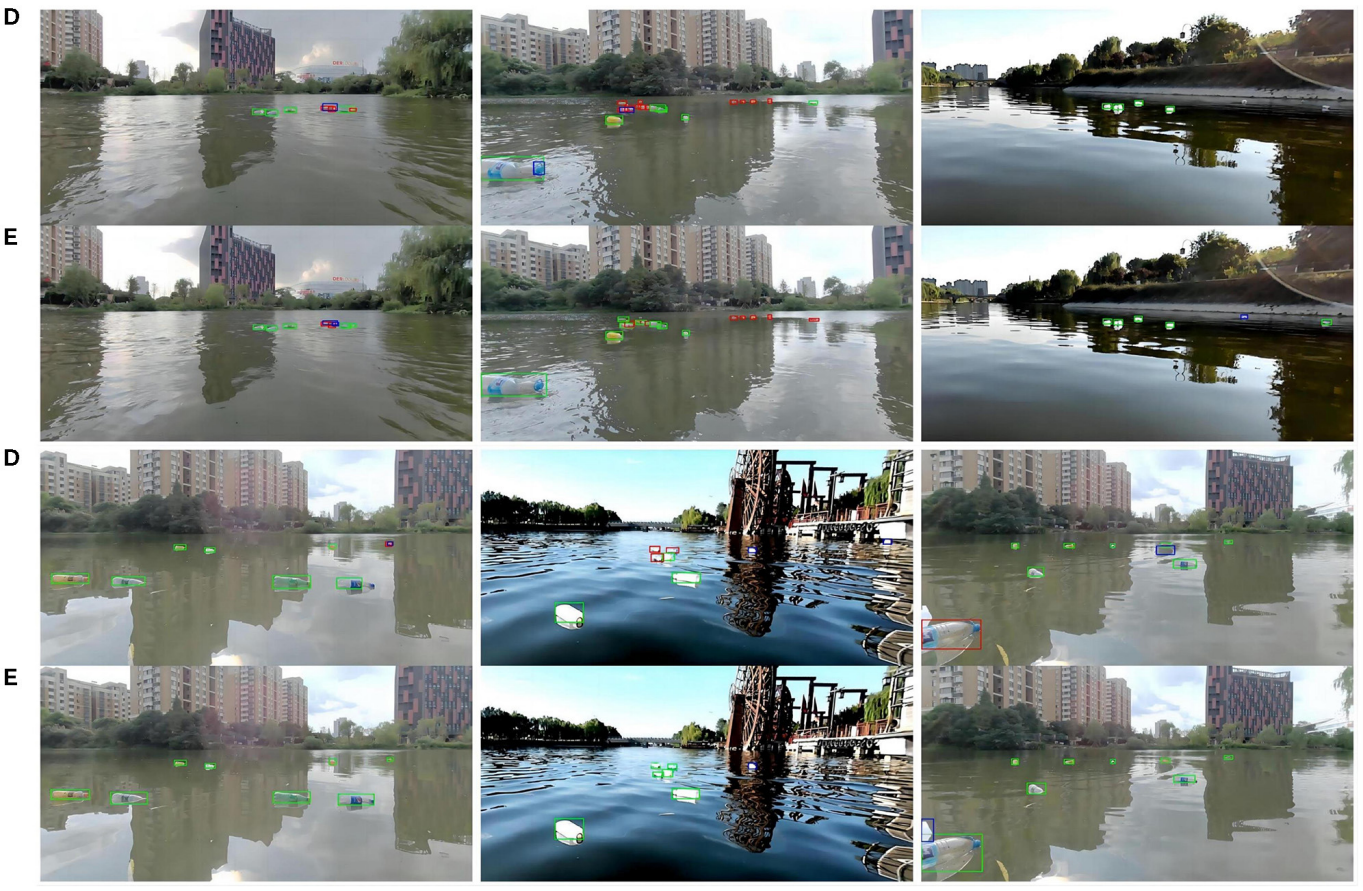
Some visualization results from our STE-YOLO on FIOW-Img. Where **(D)** represents the detection effect of the original model and **(E)** means the detection effect of STE-YOLO, it is obvious that our model has better detection effect of small targets.

To provide a visual representation of the experimental outcomes, a selection of images was curated for display purposes. As depicted in Figure 8, a compilation of heat maps and detection results is presented, offering insights into the model's performance across images featuring both large and small objects. This visualization serves to further illustrate the efficacy and versatility of the proposed approach in capturing objects of varying scales within the detection framework. As depicted in Figure 9, We also compared the detection results of different models on small targets and marked the detection situation. After testing the whole verification set, we made statistics on the detect results, as shown in Table 6. Compared to YOLOV7, STE-YOLO's error detection rate has been greatly reduced this shows that STE-YOLO has better small target detection performance.

**Table 6:** Statistics of detection results on FIOW-Img.

**Methods**	**Right**	**Miss**	**Error**
YOLOv7	1,501	368	455
STE-YOLO	1,616	288	351

## 5 Discussion

Addressing the intricate task of accurately identifying and discerning small targets within the realm of waterborne unmanned crafts' target detection, this study introduces the STE-YOLO algorithm. This algorithm represents a swift and precise technique for waterborne target detection, bolstered by enhanced small-target detection capabilities. Its primary objective is to elevate the precision of detecting small targets within this context. Achieving this aim, the algorithm integrates a pioneering P2 detection head branch that adeptly tackles the challenge of detecting targets spanning a wide range of scales. To further enhance its ability to recognize small targets, a lightweight vision center (LVC) module is seamlessly integrated to effectively synchronize cross-layer information. Simultaneously, to optimize the algorithm's computational efficiency for efficient real-world deployment, an aggregation network named L-ELAN is seamlessly woven into the backbone network architecture, thereby enhancing computational efficiency. In a strategic move to fortify the algorithm's robustness across varying scales, the Wise-IOU loss function is introduced. This dynamic loss function optimizes gradient influence arising from samples of varied quality, further enhancing model performance. Significantly, empirical evidence garnered from experiments conducted on the WSODD dataset affirms the supremacy of STE-YOLO in terms of detection accuracy, particularly its proficiency in detecting small targets, and its efficient utilization of model parameters. This advancement carries theoretical importance for the pragmatic deployment of water surface target detection applications.

As future research avenues unfold, a key focus will be refining the network structure. Promising methodologies such as model pruning or knowledge distillation will be employed to comprehensively reduce the number of network parameters. This strategic effort aims to enhance the model's applicability and performance during deployment, especially in scenarios marked by limited computational resources.

## Data availability statement

The original contributions presented in the study are included in the article/supplementary material, further inquiries can be directed to the corresponding author.

## Author contributions

JY: Writing—original draft. HZ: Writing—original draft. LX: Conceptualization, Writing—original draft. LZ: Writing—review & editing. MY: Writing—review & editing. JH: Writing—review & editing.
